# Anakinra Drug Retention Rate and Predictive Factors of Long-Term Response in Systemic Juvenile Idiopathic Arthritis and Adult Onset Still Disease

**DOI:** 10.3389/fphar.2019.00918

**Published:** 2019-08-23

**Authors:** Jurgen Sota, Donato Rigante, Piero Ruscitti, Antonella Insalaco, Paolo Sfriso, Salvatore de Vita, Rolando Cimaz, Giuseppe Lopalco, Giacomo Emmi, Francesco La Torre, Claudia Fabiani, Alma Nunzia Olivieri, Marco Cattalini, Daniele Cammelli, Romina Gallizzi, Maria Alessio, Raffaele Manna, Ombretta Viapiana, Micol Frassi, Manuela Pardeo, Armin Maier, Carlo Salvarani, Rosaria Talarico, Marta Mosca, Serena Colafrancesco, Roberta Priori, Maria Cristina Maggio, Carla Gaggiano, Salvatore Grosso, Fabrizio De Benedetti, Antonio Vitale, Roberto Giacomelli, Luca Cantarini

**Affiliations:** ^1^Research Center of Systemic Autoinflammatory Diseases and Behçet’s Disease Clinic, Department of Medical Sciences, Surgery and Neurosciences, University of Siena, Siena, Italy; ^2^Institute of Pediatrics, Fondazione Policlinico A. Gemelli IRCCS, Rome, Italy; ^3^Periodic Fever Research Center, Università Cattolica Sacro Cuore, Rome, Italy; ^4^Division of Rheumatology, Department of Biotechnological and Applied Clinical Science, University of L’Aquila, L’Aquila, Italy; ^5^Division of Rheumatology, Department of Pediatric Medicine, Bambino Gesù Children’s Hospital IRCCS, Rome, Italy; ^6^Rheumatology Unit, Department of Medicine, University of Padua, Padua, Italy; ^7^Department of Medical and Biological Sciences, Rheumatology Clinic, University of Udine, Udine, Italy; ^8^Rheumatology Unit, Meyer Children’s Hospital, University of Florence, Florence, Italy; ^9^Department of Emergency and Organ Transplantation-Rheumatology Unit, University of Bari, Bari, Italy; ^10^Department of Experimental and Clinical Medicine, University of Florence, Florence, Italy; ^11^Pediatric Rheumatology Section, Pediatric Unit, Giovanni XXIII - Pediatric Hospital, Bari, Italy; ^12^Ophthalmology Unit, Department of Medicine, Surgery and Neuroscience, University of Siena, Siena, Italy; ^13^Dipartimento della donna del bambino e di chirurgia generale e specialistica, Università della Campania Luigi Vanvitelli, Italy; ^14^Pediatric Clinic, University of Brescia and Spedali Civili di Brescia, Brescia, Italy; ^15^Experimental and Clinical Medicine Department, University of Florence, Florence, Italy; ^16^Department of Pediatrics, Azienda G. Martino, University of Messina, Messina, Italy; ^17^Department of Pediatrics, University Federico II, Naples, Italy; ^18^Rheumatology Section, Department of Medicine, University of Verona, Verona, Italy; ^19^Rheumatology and Clinical Immunology Unit, Department of Clinical and Experimental Sciences, University of Brescia and Spedali Civili di Brescia, Brescia, Italy; ^20^Struttura Semplice di Reumatologia, Ospedale di Bolzano, Bolzano, Italy; ^21^Rheumatology Unit, Department of Internal Medicine, Azienda Ospedaliera ASMN IRCCS, Reggio Emilia, Italy; ^22^Rheumatology Unit, Department of Clinical and Experimental Medicine, University of Pisa, Pisa, Italy; ^23^Department of Internal Medicine and Medical Specialties, Rheumatology Unit, Sapienza University of Rome, Rome, Italy; ^24^Universitary Department “Pro.S.A.M.I.”, University of Palermo, Palermo, Italy; ^25^Clinical Pediatrics, Department of Molecular Medicine and Development, University of Siena, Siena, Italy

**Keywords:** anakinra, interleukin 1-beta, innovative biotechnologies, drug retention rate, systemic juvenile idiopathic arthritis, adult onset Still disease, personalized medicine

## Abstract

**Background and Objective:** Only a few studies have reported long-term efficacy of interleukin (IL)-1 inhibition in systemic juvenile idiopathic arthritis (sJIA) and adult-onset Still disease (AOSD). Herein we report on the effectiveness of anakinra (ANA), expressed in terms of drug retention rate (DRR), and evaluate the predictive factors of drug survival in a cohort of patients with sJIA and AOSD.

**Patients and Methods:** This is a multicenter study reviewing retrospectively the medical records from 61 patients with sJIA and 76 with AOSD, all treated with ANA in 25 Italian tertiary referral centers.

**Results:** The cumulative retention rate of ANA at 12-, 24-, 48-, and 60-month of follow-up was 74.3%, 62.9%, 49.4%, and 49.4%, respectively, without any significant differences between sJIA and AOSD patients (*p* = 0.164), and between patients treated in monotherapy compared with the subgroup coadministered with conventional disease-modifying antirheumatic drugs (cDMARDs) (*p* = 0.473). On the other hand, a significant difference in DRR was found between biologic-naïve patients and those previously treated with biotechnologic drugs (*p* = 0.009), which persisted even after adjustment for pathology (*p* = 0.013). In the regression analysis, patients experiencing adverse events (AEs) {hazards ratio (HR) = 3.029 [confidence interval (CI) 1.750–5.242], *p* < 0.0001} and those previously treated with other biologic agents [HR = 1.818 (CI 1.007–3.282), *p* = 0.047] were associated with a higher HR of ANA discontinuation. The median treatment delay was significantly higher among patients discontinuing ANA (*p* < 0.0001). Significant corticosteroid-sparing (*p* = 0.033) and cDMARD-sparing effects (*p* < 0.0001) were also recorded. Less than one-third of our cohort developed AEs, and 85% were deemed mild in nature, with 70% of them involving the skin.

**Conclusions:** Our findings display an overall excellent DRR of ANA on the long run for both sJIA and AOSD, that may be further optimized by closely monitoring patient’s safety issues and employing this IL-1 inhibitor as a first-line biologic as early as possible. Moreover, ANA allowed a significant drug-sparing effect and showed an overall good safety profile.

## Introduction

Systemic juvenile idiopathic arthritis (sJIA) and adult-onset Still disease (AOSD) represent two multifactorial nonhereditary autoinflammatory disorders related to pediatric and adult patients, respectively, characterized by a defective control of innate immunity, as depicted for hereditary autoinflammatory disorders, and by daily high spiking fevers along with systemic features including serositis, evanescent rash, generalized lymphoadenopathy, and arthritis (Caso et al., 2014; Cimaz, 2016; Rigante, 2017; Giacomelli et al., 2018; Lyseng-Williamson, 2018). These two conditions are accompanied by a relevant risk of mortality (Priori et al., 2010; Kimura et al., 2013; Minoia et al., 2014; Kumar, 2016; Giacomelli et al., 2018), making a timely diagnosis as well as a prompt treatment mandatory. Treatment of both sJIA and AOSD has traditionally relied on nonsteroidal anti-inflammatory drugs, corticosteroids (CS), and conventional disease-modifying antirheumatic drugs (cDMARDs), with methotrexate being the most frequently used (Gerfaud-Valentin et al., 2014; Kumar, 2016). However, there is limited evidence, especially for sJIA, regarding other effective drugs, such as cyclosporine, thalidomide, and other novel agents (Kumar, 2016; Mauro et al., 2017). The lack of standardized therapeutic guidelines represents an important unmet need, and management of both sJIA and AOSD still remains empirical. Biotechnological drugs have also proved to be effective in AOSD (Ruscitti et al., 2017), and indeed many compelling pathogenetic data (Pascual et al., 2005; Church et al., 2008; Mellins et al., 2011; Gerfaud-Valentin et al., 2014) consider interleukin (IL)-1 as the main orchestrating cytokine in sJIA and AOSD pathways, providing the biologic rationale of IL-1 inhibition in these two entities, which are now deemed *autoinflammatory* in nature (Hayem, 2009; Rossi-Semerano and Koné-Paut, 2012).

Anakinra (ANA) is a recombinant human IL-1 receptor “antagonist,” which binds tightly to the IL-1 receptor and competitively prevents activation of this receptor by either IL-1α and IL-1β (Mistry et al., 2017). After showing its paramount efficacy in the cryopyrin-associated periodic syndrome, a rare IL-1-mediated hereditary autoinflammatory disorder (Cantarini et al., 2011; Rigante, 2018), ANA started to be employed also in AOSD and sJIA. From its first use on AOSD (Rudinskaya and Trock, 2003) and sJIA (Verbsky and White, 2004), a growing body of evidence has reported the considerable clinical efficacy of ANA in both entities (Quartier et al., 2011; Nordström et al., 2012; Sfriso et al., 2016; Colafrancesco et al., 2017) with several studies suggesting this targeted biologic therapy as first- or early second-line treatment (Pascual et al., 2005; Moulis et al., 2010; Nigrovic et al., 2011; Hedrich et al., 2012; Vastert et al., 2014; Pardeo et al., 2015). Nevertheless, only a few data are available in regard to long-term efficacy and tolerability of ANA in sJIA and AOSD (Lequerré et al., 2008; Laskari et al., 2011; Giampietro et al., 2013; Ortiz-Sanjuán et al., 2015; Colafrancesco et al., 2017).

Herein, we report a multicenter real-life experience on patients with sJIA and AOSD in the long term, evaluating ANA effectiveness in terms of drug retention rate (DRR) along with predictive factors associated with treatment withdrawal.

## Patients and Methods

### Study Design and Participants

Medical records of 61 patients affected by sJIA and 76 patients affected by AOSD, all treated with ANA and enrolled from January 2008 until July 2016 in 25 Italian tertiary rheumatology referral centers, were retrospectively reviewed. Clinical and therapeutic data were collected in combination with general and demographic data, such as age, gender, age at disease onset, disease duration, treatment delay, the anti-IL-1 agent employed, dosages used, concomitant and previous treatments, and overall anti-IL-1 treatment duration.

sJIA was diagnosed according to the revised International League of Association for Rheumatology (ILAR) criteria (Petty et al., 2004), while AOSD diagnosis was established according to the Yamaguchi criteria (Yamaguchi et al., 1992). In accordance with the best standards of care, all patients were systematically followed-up every 3 months and/or in case of necessity (disease flare and/or safety issues). Before starting anti-IL-1 treatment with ANA, patients underwent a complete medical examination and an extensive work-up for infectious diseases, including search for markers of hepatitis B and C viruses, urine culture, QuantiFERON test, and chest X-ray to rule out active or latent infections. ANA dosages ranged from 1 to 4 mg/kg/day for pediatric patients, while 100 mg/day was the starting dose for adult patients.

### Aims and Endpoints

Primary aim of the study was to examine the overall DRR of ANA in sJIA and AOSD patients. Secondary aims were to: (i) explore the influence of the biologic line of treatment and the concomitant use of cDMARDs on DRR in the whole sample and stratified according to the disease thereafter; (ii) find eventual predictive factors associated with events leading to drug discontinuation. The CS- and cDMARDS-sparing effects, impact of treatment delay on survival, and record of safety profile were considered ancillary aims of the study.

The primary endpoint was evaluated by the Kaplan–Meier survival curve at 12, 24, 48, and 60 months of follow-up. Secondary endpoints were as follows: (i) using limit estimators to compare survival curves of monotherapy *versus* combination therapy with cDMARDs and significant differences on survival curves, distinguishing between biologic-naïve patients and those already treated with other biologics; the analysis was then extended by stratifying limit estimators according to the disease; (ii) to evaluate whether demographic, clinical, and therapeutic variables could predict time-to-treatment discontinuation. Finally, ancillary aims were explored by the identification of potential statistically significant differences in the mean treatment delay, subdividing our sample in patients continuing and patients discontinuing treatment as well as on CS- and cDMARDs-sparing effect, and description of AEs.

### Statistical Analysis

Data were analyzed using IBMSPSS Statistics for Windows, version 24 (IBM Corp., Armonk, NY, USA). Descriptive statistics was used to display mean and standard deviation (SD) or median and interquartile ranges (IQRs), as required. We analyzed data distribution with the Shapiro–Wilk test. Categorical variables were analyzed by McNemar test for repeated measures, while differences in means were investigated with Mann–Whitney *U* test. Time-to-event analysis was performed according to the Kaplan–Meier method, with the event being ANA discontinuation. Patients discontinuing ANA due to remission were not included in the statistical analysis. Survival curves were compared using both long-rank and Breslow test as limit estimators. Event-free survival was assessed with a Cox proportional hazard model, using 95% confidence interval (CI) for hazard ratio (HR) aiming to evaluate any relation of demographic, clinical, and therapeutic data with DRR. The threshold for statistical significance was set to *p* < 0.05, and all *p* values were two-sided.

## Results

We examined medical charts of 137 patients (56 males, 81 females) affected by sJIA (61 patients) and AOSD (76 patients), all receiving ANA within the study period lasting from January 2008 to July 2016.

Demographic, clinical, and therapeutic data of sJIA and AOSD are detailed in **Tables 1** and **2**, respectively. Female-to-male ratio was 1.03 for sJIA patients and 1.92 for the AOSD ones. The median ± IQR time of treatment duration was 18.00 ± 27.00 months. The mean ± SD age at disease onset for AOSD was 39.98 ± 15.05, while the median age ± IQR at disease onset for sJIA was 5.80 ± 7.10 years. Forty-two out of 137 patients were coadministered with cDMARDs, and 25 subjects had been previously treated with other biologic agents.

**Table 1:** Demographic and therapeutic features of patients with sJIA.

Patients no.	61
Male/Female no.	30/31
Mean age ± SD (years)	12.97 ± 7.05
Age at onset (median ± IQR) (years)	5.80 ± 7.10
Age at diagnosis (median ± IQR) (years)	6.70 ± 7.30
Disease duration (median ± IQR) (years)	4.00 ± 5.93
Treatment delay (median ± IQR) (years)	2.10 ± 6.64
Previous biologics	ETN (n = 9); IFX (n = 1); ADA (n = 1); TCZ (n = 2); CAN (n = 1); RTX (n = 1); ABA (n = 1).
Previous cDMARDs	MTX (n = 14); CsA (n = 13); SSZ (n = 2); LFN (n = 2); HCQ (n = 2)
Concomitant cDMARDs	MTX (n = 8); CsA (n = 8); SSZ (n = 1); LFN (n = 1); HCQ (n = 2)

ABA, abatacept; ADA adalimumab; CAN, canakinumab; CsA cyclosporine; ETN, etanercept; HCQ, hydroxychloroquine; IFX, infliximab; LFN, leflunomide; MTX, methotrexate; RTX, rituximab; SSZ, sulfasalazine; TCZ, tocilizumab; sJIA, systemic juvenile idiopathic arthritis; IQR, interquratile range; cDMARDs, conventional disease modifying anti-rheumatic drugs.

**Table 2:** Demographic and therapeutic data of patients with AOSD.

Patients no.	76
Male/Female no.	26/50
Mean age ± SD (years)	47.90 ± 15.13
Age at onset (mean ± SD) (years)	39.98 ± 15.05
Age at diagnosis (mean ± SD) (years)	41.96 ± 14.49
Disease duration (median ± IQR) (years)	6.00 ± 9.00
Treatment delay (median ± IQR) (years)	3.25 ± 7.47
Previous biologics	ETN (n = 9); IFX (n = 6); ADA (n = 3); GOL (n = 1); TCZ (n = 1); RTX (n = 1)
Previous cDMARDs	MTX (n = 42); CsA (n = 18); SSZ (n = 4); LFN (n = 2); HCQ (n = 19); AZA (n = 4); CPH (n = 1); CQN (n = 2); COL (n = 2).
Concomitant cDMARDs	MTX (n = 18); CsA (n = 4); SSZ (n = 1); LFN (n = 2); HCQ (n = 7)

ADA, adalimumab; AZA, azathioprine; CQN, chloroquine; COL, colchicine; CPH, cyclophosphamide; CsA, cyclosporine; ETN, etanercept; GOL, golimumab; HCQ, hydroxychloroquine; IFX, infliximab; LFN, leflunomide; MTX, methotrexate; RTX, rituximab; SSZ, sulfasalazine; TCZ, tocilizumab AOSD, Adult onset Still disease; IQR, interquartile range; cDMARDs, conventional disease modifying anti-rheumatic drugs.

**Figure 1:**
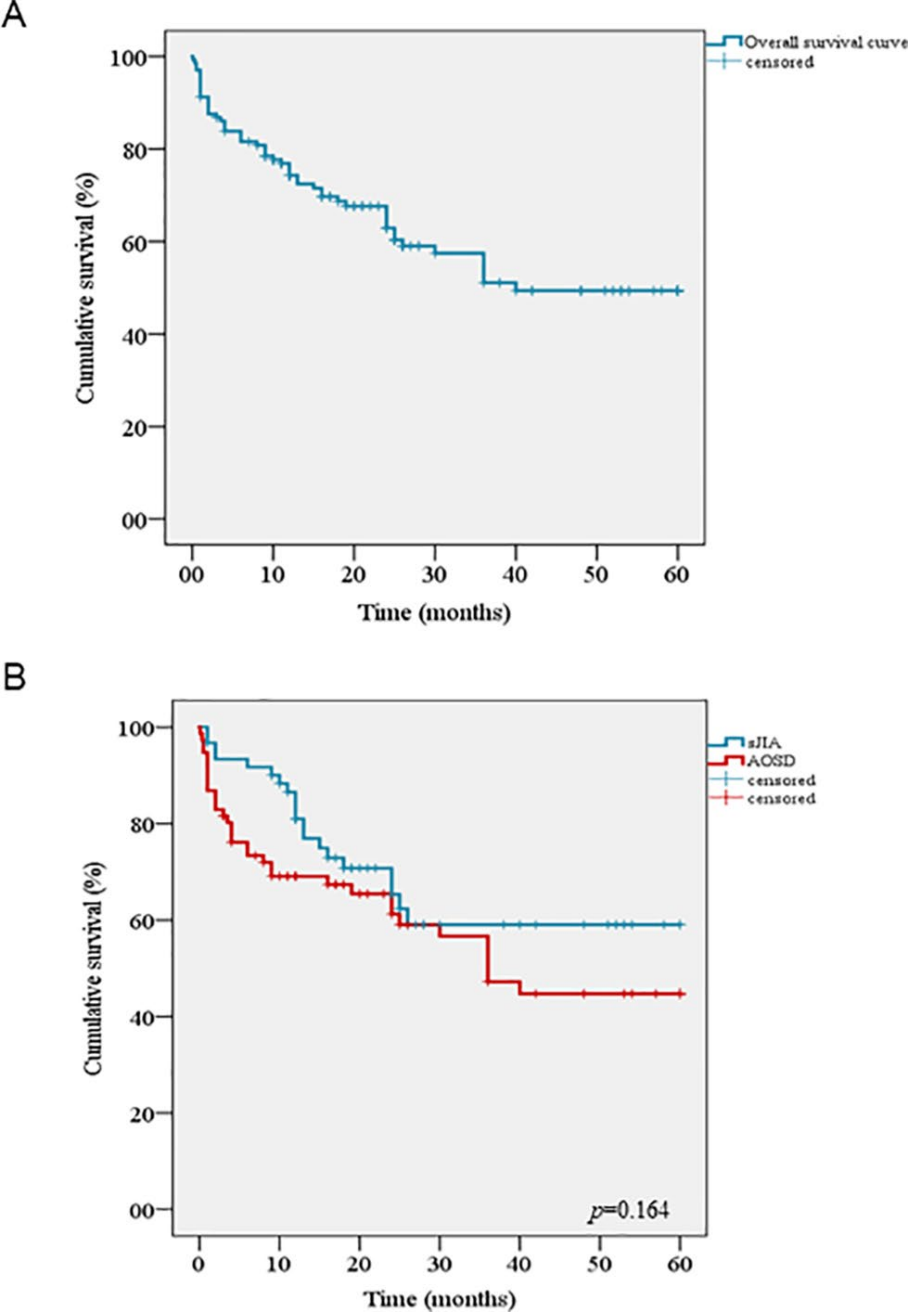
Kaplan–Meier curves describing the cumulative survival of Anakinra related to: **(A)** the entire cohort of patients, **(B)** the log rank test comparing drug survival in systemic juvenile idiopathic arthritis and adult onset Still disease.

**Figure 2:**
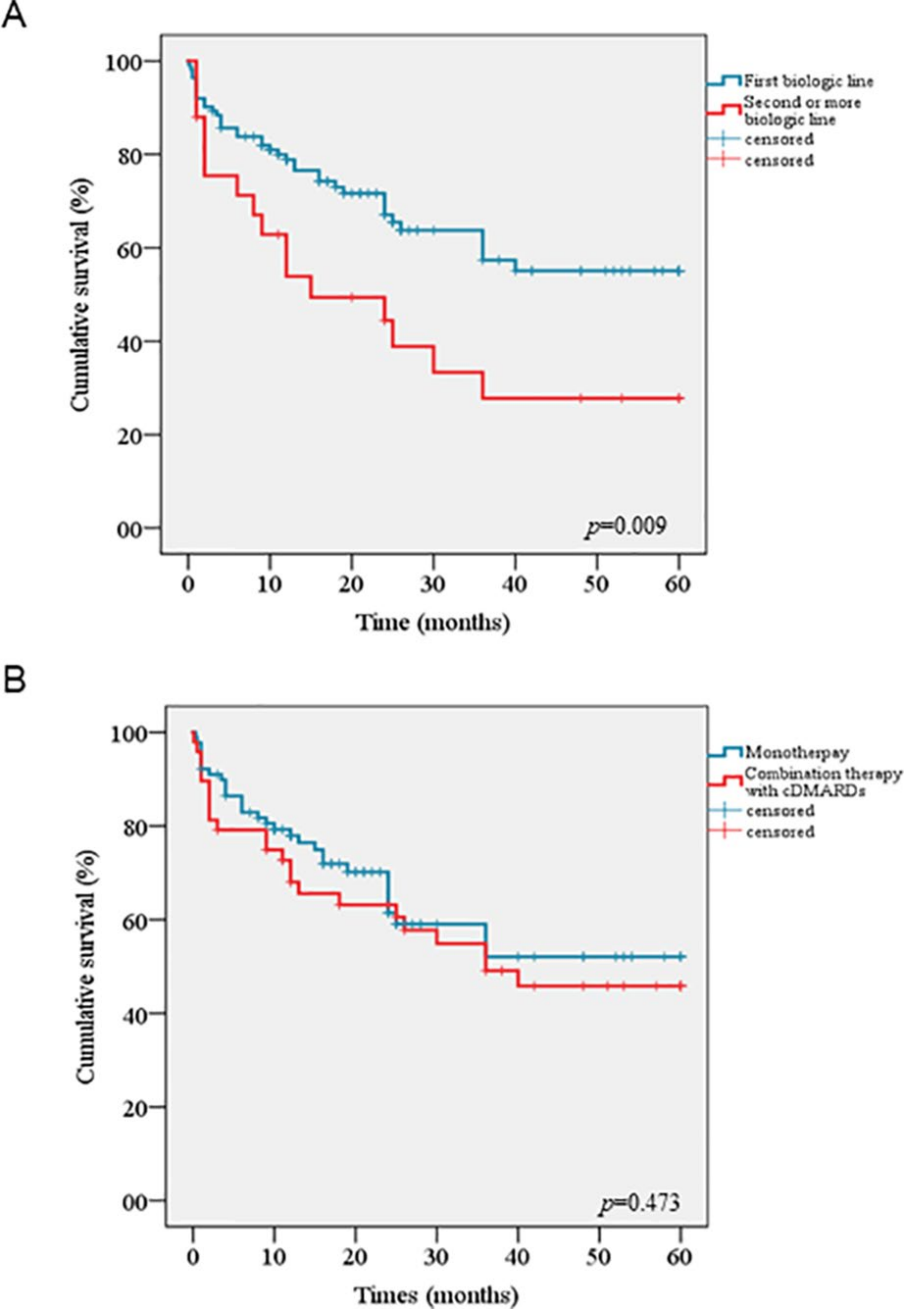
Kaplan–Meier curves comparing cumulative drug survival of Anakinra between biologic-naïve patients and those pretreated with other biologics **(A)**, between patients undergoing monotherapy and the subgroup coadministered with conventional disease modifying anti-rheumatic drugs **(B)**.

The cumulative retention rate of ANA at 12, 24, 48, and 60 months of follow-up was 74.3%, 62.9%, 49.4%, and 49.4%, respectively (**Figure 1A**), without any significant differences between sJIA and AOSD patients (*p* = 0.164) (**Figure 1B**). Conversely, statistically significant differences were observed between biologic-naïve patients and those previously treated with other biologic drugs (*p* = 0.009) (**Figure 2A**). The difference between the two subgroups persisted also after adjustment for pathology (*p* = 0.013). In addition, no statistically significant differences were detected between patients in monotherapy and the subgroup coadministered with cDMARDs (*p* = 0.473) (**Figure 2B**). Cox regression analysis identified two variables associated with a higher hazard of treatment withdrawal: line of biologic treatment and AEs. More in detail, patients previously treated with other biologics displayed a higher HR [HR = 1.818 (CI 1.007–3.282), *p* = 0.047]. Similarly, the subgroup experiencing the occurrence of AEs was also associated with a higher hazard of treatment discontinuation [HR = 3.029 (CI 1.750–5.242), *p* < 0.0001].

Moreover, the median treatment delay was significantly higher among patients discontinuing ANA (4.00 ± 6.83 years) compared to the subgroup that was able to retain it (0.66 years ± 3.24) (*p* < 0.0001). With regard to the CS-sparing effect, a significant reduction in the number of patients requiring the support of CS was found (*p* = 0.033). Fifteen patients were able to interrupt CS therapy. A significant cDMARDs-sparing effect was also observed (*p* < 0.0001) and 35 patients were able to discontinue treatment with immunosuppressive drugs.

AEs occurred in 40 out of 137 patients (29.2%) (11 in sJIA and 29 in AOSD patients), with the most frequent being injection site reactions (n = 18), followed by generalized skin rashes (n = 10), flue-like syndrome (n = 2), thrombocytopenia (n = 2), transient mild respiratory problems (n = 1), and abnormal level of liver enzymes (n = 1). With regard to serious AEs, two cases of pneumonia along with lower limb ulcers (n = 1), myocarditis (n = 1), dilated cardiomyopathy (n = 1), and macrophage activation syndrome (MAS) (n = 1) were recorded. Death occurred in four patients experiencing serious AEs (two patients with pneumonia and the remaining two suffering from myocarditis and dilatative cardiomyopathy, respectively). Overall, AEs caused treatment discontinuation in 29 cases. The remaining causes for discontinuing treatment were as follows: clinical remission (n = 25), lack of efficacy (n = 8), loss of efficacy (n = 15), and pregnancy (n = 1). AEs are summarized in **Table 3**.

**Table 3:** Adverse events recorded for sJIA and AOSD.

sJIA	N
Injections site reaction	7
Generalized skin rash	2
Hepatic toxicity	1
Transient and mild breath problems	1
AOSD	N
Injection site reaction	11
Generalized skin rash	8
Thrombocytopenia	2
Flu-like syndrome	2
Pneumonia	2
Lower limb ulcers	1
Myocarditis	1
Dilated cardiomyopathy	1
macrophage activation syndrome	1

sJIA, systemic juvenile idiopathic arthritis; AOSD, Adult onset Still disease.

## Discussion

sJIA is the pediatric counterpart of AOSD and both disorders represent hard-to-handle autoinflammatory conditions characterized by high mortality rate due to the risk of severe fatal complications (Kimura et al., 2013; Minoia et al., 2014; Lopalco et al., 2015; Kumar, 2016; Ruscitti et al., 2016; Giacomelli et al., 2018). Additionally, a high percentage of patients with sJIA and AOSD can be refractory to CS, conventional immunosuppressants, and also several biologic agents (Nordström et al., 2012; Ortiz-Sanjuán et al., 2015; Giancane et al., 2016; Janow et al., 2016). Nevertheless, in the current biologic era, novel targeted treatments have favorably resized the therapeutic armamentarium for these two entities. Several cytokines, such as IL-1 and IL-18, processed through the inflammasome machinery, and also IL-6 have been implicated in their pathogenesis. Particularly, since IL-1 was considered the master mediator of inflammatory pathway in both sJIA and AOSD (Pascual et al., 2005; Church et al., 2008; Mellins et al., 2011; Gerfaud-Valentin et al., 2014), an increasing experience of IL-1 blockade has matured over time (Lequerré et al., 2008; Nigrovic et al., 2011; Quartier et al., 2011; Nordström et al., 2012; Ortiz-Sanjuán et al., 2015; Pardeo et al., 2015; Sfriso et al., 2016; Colafrancesco et al., 2017).

In the present study we have highlighted the real-life experience of 25 Italian tertiary referral centers with the recombinant human IL-1 receptor ANA in the treatment of sJIA and AOSD, focusing on long-term effectiveness as well as on any potential differences between the two disorders. The study involved 61 sJIA patients and 76 ASOD patients. In the sJIA subgroup males and females were equally represented, whereas AOSD sample was skewed toward females, who composed 65.8% of the population. This is in accordance with previous studies reporting almost exactly the same percentage (Sakata et al., 2016; Mehta et al., 2019).

Our findings suggest an overall excellent DRR of ANA in both sJIA and AOSD, with an estimated probability of 50% to persist on treatment after 5 years from its initiation. The DRR of ANA does not significantly differ between patients with sJIA and AOSD, implying a similar effectiveness of ANA for both disorders. The drug survival of ANA is not affected by the concomitant use of cDMARDs, highlighting the effectiveness of this specific drug when employed in monotherapy. Other studies reporting long-term efficacy of ANA in sJIA and AOSD patients had shown similar results when comparing adjunct therapy *versus* monotherapy in terms of survival analysis as well as clinical and laboratory response (Laskari et al., 2011, Vitale et al., 2019). Data reported in this study revealed the similar effectiveness of ANA in sJIA and AOSD, though it remains controversial whether sJIA and AOSD could be considered as an identical disease. However, based on these successful results, many experts contemplate these two entities as a *continuum* of one only disorder affecting different onset ages (Hayem, 2009; Nirmala et al., 2015; Junge et al., 2017; Lyseng-Williamson, 2018; Martini et al., 2019).

On the other hand, ANA DRR differs significantly between biologic-naïve patients and those already treated with other biotechnologic agents. This observation is in accordance with previous studies recommending ANA administration as first-line biologic agent instead of a rescue therapy in sJIA (Nigrovic et al., 2011; Hedrich et al., 2012; Vastert et al., 2014; Pardeo et al., 2015). In addition, this option might also be applied to AOSD, since the difference between biologic-naïve and biologic-exposed patients still persisted also on a stratified analysis after adjustment for disorder. As also shown by the regression analysis, the biologic line of treatment seems to be a predictive factor for treatment withdrawal with a significantly lower HR for biologic-naïve patients.

Another crucial issue is related to the demand for a prompt introduction of cytokine-blocking therapies to modify the natural disease course by preventing or reducing any structural damage secondary to long-term improperly controlled disease. The significant higher mean treatment delay in patients discontinuing ANA advocates for a timely establishment of biologic therapy. In fact, the therapeutic potential of IL-1 blockade may be fully explored when ANA is used within the “windows of opportunity,” an interesting concept firstly developed in 2014 (Nigrovic, 2014). Indeed, early treatment with IL-1 blockade is presumably able to alter disease progression of both sJIA and AOSD by slowing its evolution and avoiding permanent damages. On the other hand, it is also possible that patients with long-standing disease receiving multiple biologic agents may simply have a more resistant disease. Timely targeted treatment is also important for minimizing inappropriately high cumulative dose of CS and their associated detrimental side effects. Concordant with other authors’ observations (Lequerré et al., 2008; Laskari et al., 2011; Giampietro et al., 2013), in our sample, we found a CS-sparing effect with a significant lower number of patients requiring CS administration on the last follow-up visit. Noteworthy, Nigrovic et al. found that the majority of patients were CS-free by 2 months (Nigrovic et al., 2011). Interestingly, a cDMARDs-sparing effect was also observed. The use of IL-1 inhibition has even reduced side effects related to cotreatments while increasing patient’s compliance to monotherapy (Giampietro et al., 2013). This drug-sparing effect is essential in the setting of chronic systemic inflammatory disorders in the light of the reduction of side effects, which is of utmost relevance in the pediatric age.

As for AEs, we observed a good overall safety profile for ANA, as only less than one-third of the patients developed AE, which were mild in the vast majority of cases. More in detail, 70% of AEs involved the skin, including injection site reactions in 18 cases. Four cases of death occurred in our cohort of patients with AOSD. However, these cases are considered to be more likely related to the underlying preexisting comorbidities and/or the poor clinical condition of patients with a refractory or complicated AOSD (Kim et al., 2012; Kimura et al., 2013; Minoia et al., 2014; Kumar, 2016; Giacomelli et al., 2018). Additionally, only one patient with AOSD and none of patients with sJIA developed MAS during the follow-up period. In agreement with the latter, a positive experience with ANA in treating pediatric patients with MAS has been also reported (Sönmez et al., 2018). Besides their single center experience showing the resolution of clinical pictures as well as normalization of laboratory parameters, Sönmez et al. performed a systematic literature review and found optimal results in terms of remission and safety profile of ANA in the treatment of MAS. On the other hand, controversial results have been reported with other anti-IL-1 agents (Ilowite et al., 2014; Grom et al., 2016). Only one episode of MAS occurred in the long-term extension phase of a randomized study investigating efficacy and safety of rilonacept in sJIA patients (Ilowite et al., 2014). Contrarily, Grom et al. stated that MAS occurs even in sJIA patients properly controlled with biologic therapy (Grom et al., 2016). Hence, it is not possible to draw firm conclusions and dedicated prospective randomized studies specifically investigating this dreadful complication are warranted to shed light on this topic.

Potential limitations of our study is its retrospective noncomparative design. Data such as laboratory markers and Pouchot’s score were not collected, and therefore we could not assess whether they predicted treatment withdrawal or not. Additionally, given the absence of standardized treatment guidelines, the management of the single patient relied on the personal experience of the local physician.

## Conclusions

ANA appears a promising drug in both sJIA and AOSD, also when the disease has a long-standing course, displaying a satisfying clinical effectiveness and minimizing long-term requirement of CS. Moreover, ANA exhibits a similar DRR in both sJIA and AOSD and their survival is not affected by the concomitant use of cDMARDs, highlighting its efficacy as monotherapy. A tight monitoring of safety profile is also mandatory, since it can significantly affect ANA DRR. Additionally, DRR is also significantly influenced from the biologic line of treatment, suggesting to employ ANA as a first-line biologic agent. Lastly, our results reveal a significant impact of treatment delay in drug discontinuation, implying that a trend toward an earlier initiation might be crucial to take advantage of the “windows of opportunity” and improve long-term outcomes of patients with both sJIA and AOSD.

## Data Availability

The datasets generated for this study are available on request to the corresponding author.

## Ethics Statement

This study adhered to the tenets of the Declaration of Helsinki, and the protocol was approved by the local Ethic Committee (Meyer Children's Hospital, reference number: 364-16OCT2013). Informed written consent was obtained from each patient or their legal guardians prior to being enrolled.

## Author Contributions

JS, DR, and LC designed the study. JS performed the statistical analysis. JS and LC wrote the first draft of the manuscript, DR revised the final draft. All other authors, including PR, AI, PS, SdV, RC, GL, GE, FLT, CF, ANO, MC, CD, RGa, MA, RM, OV, MF, MP, AM, CS, RT, MM, SC, RP, MCM, CG, SG, FDB, AV, RGi have critically reviewed the final draft of the manuscript and approved the submitted version.

## Conflict of Interest Statement

The authors declare that the research was conducted in the absence of any commercial or financial relationships that could potentially be construed as a conflict of interest.
